# Sensing mycobacteria through unconventional pathways

**DOI:** 10.1172/JCI190230

**Published:** 2025-03-17

**Authors:** Catarina F. Almeida, Jennifer A. Juno

**Affiliations:** Department of Microbiology and Immunology, University of Melbourne at the Peter Doherty Institute for Infection and Immunity, Melbourne, Australia.

## Abstract

Approximately one-quarter of the global population is estimated to be infected with *Mycobacterium tuberculosis*. New developments in vaccine design and therapeutics are urgently needed, particularly in the face of multidrug-resistant tuberculosis (TB). In this issue of the *JCI*, Sakai and colleagues used a multidisciplinary approach to determine that trehalose-6-monomycolate (TMM), a mycobacterial cell wall lipid, serves as a T cell antigen presented by CD1b. CD1b-TMM–specific T cells were characterized by conserved T cell receptor features and were present at elevated frequencies in individuals with active TB disease. These findings highlight the dual role of TMM in stimulating both innate and adaptive immunity and broaden our understanding of CD1-mediated lipid recognition by unconventional T cells.

## Immunogenic components of the mycobacterial cell wall

**Figure 1:**
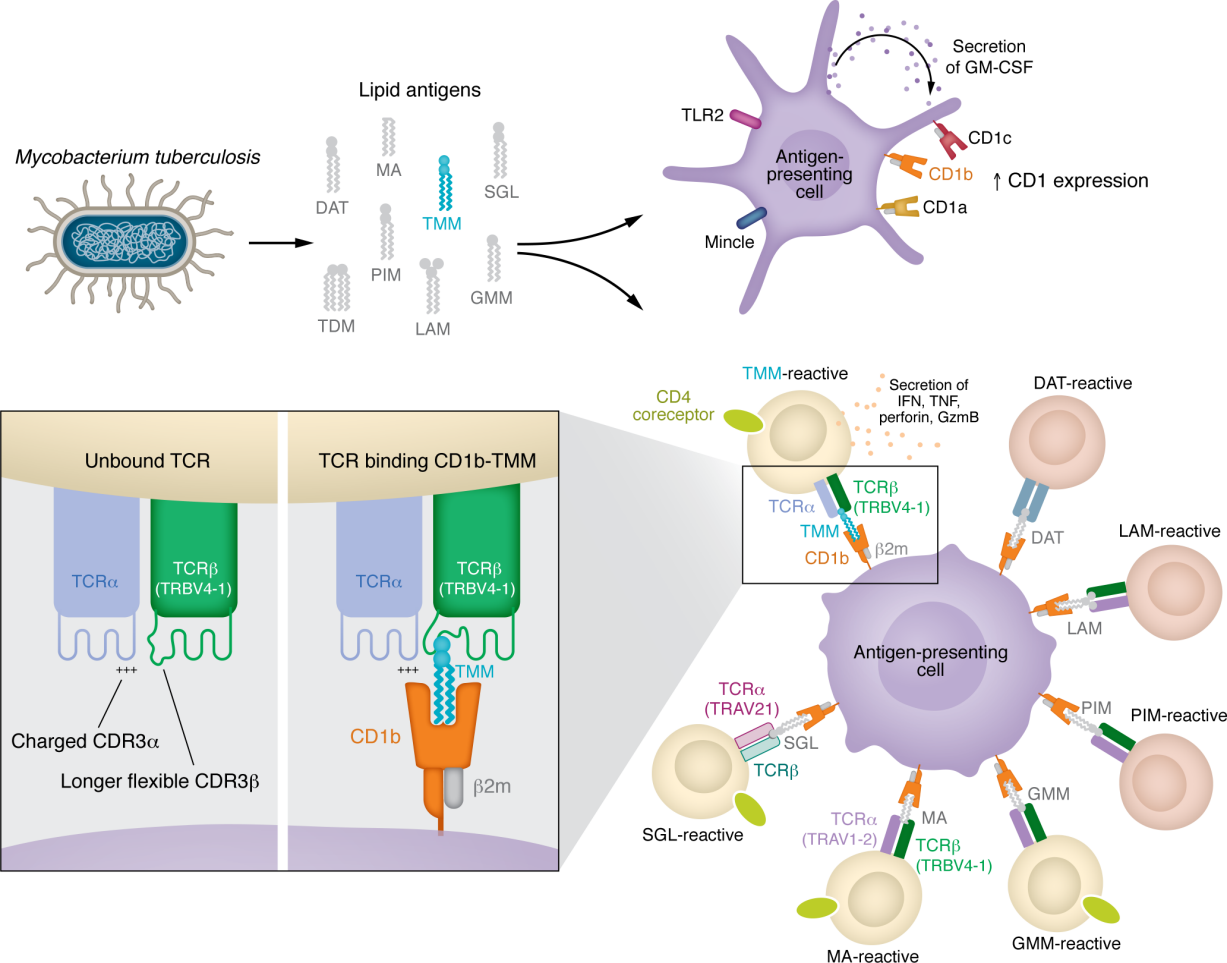
Mycobacterial lipids play a dual role in stimulating the immune system. The mycobacterial cell wall contains numerous MAs with immunostimulatory activity. Mycolate lipids such as TMM, TDM, and diacyltrehalose (DAT) bind to the C-type lectin receptor Mincle on myeloid cells, stimulating downstream inflammatory processes. Others such as LAM and PIM bind TLR2, which triggers GM-CSF secretion and CD1 upregulation on antigen-presenting cells, including myeloid cells, monocytes, and macrophages. These and other lipid-based molecules, such as MA or its ester derivatives GMM, DAT, and SGL, can also be captured by CD1b and presented to unconventional T cells that recognize such CD1b-lipid complexes via their TCR. The CD1b-TMM–specific T cell populations revealed by Sakai and colleagues (3) expand upon *M. tuberculosis* infection and exhibited conserved features shared across different individuals. Some of these characteristics are also common among other CD1b-restricted T cell subsets, including expression of the CD4 coreceptor and cytotoxicity-associated effector molecules, such as IFN, TNF, granzyme B (GzmB), and perforin. They also share a previously described TRBV4-1 usage for CD1b-restricted cells (albeit with long and flexible CDR3β loops to accommodate a complex lipid headgroup that protrudes from CD1b) and positively charged amino acids comprising the CDR3α (similar to CD1b-GMM–reactive cells), which define the TCR specificity for CD1b-TMM.

The mycobacterial cell wall contains various components including proteins, lipids, and LPS-like molecules that can stimulate the human immune system. Although the potent antigenicity and adjuvant activity of crude mycobacterial emulsions containing these components (such as CFA) are well described, many targets and mechanisms of action remain incompletely defined. Mycobacterial lipid–based molecules, in particular, can act through multiple pathways, rapidly activating innate immunity via pattern recognition receptors (PRRs) (1), as well as initiating adaptive immune responses via presentation to CD1-restricted T cells (Figure 1). The latter often involves abundant components of the mycobacterial cell wall such as mycolic acid (MA) and its ester derivatives, including glucose monomycolate (GMM), lipoarabinomannan (LAM), phosphatidylinnositolmannosides (PIM), or sulfoglycolipids (SGL) presented by CD1b, dideoxymycobactins (DDM) presented by CD1a, phosphomycoketide (PM) and mannosyl-β1-phosphomycoketide (MPM) presented by CD1c, as well as diacylglycerols and PIMs presented by CD1d (2).

In this issue of the *JCI*, Sakai and colleagues (3) reveal that trehalose-6-monomycolate (TMM), in addition to a previously described immune activation pathway that involves binding to the C-type lectin receptor Mincle (4), can also bind CD1b and stimulate T cells. Critically, Sakai and authors developed tools to reveal and characterize a public subset of unconventional T cells with conserved T cell receptor (TCR) features across different individuals that recognize CD1b-TMM complexes and accumulate upon *Mycobacterium* *tuberculosis* infection (3).

## A known lipid acts as a CD1 antigen

Using PBMCs from healthy donors, the authors performed single-cell RNA-Seq (scRNA-Seq) and TCR profiling to characterize T cell clones that proliferated in response to *M. tuberculosis* cell wall lipid extracts. Of 52 selected TCR clonotypes, 44 were successfully expressed in a retrovirus-transduced reporter system, and 1 clone, termed Y-50 (*TRAV14/DV4/TRAJ5–TRBV4-1/TRBJ2-3*), clearly exhibited TCR specificity to *M. tuberculosis* lipid extracts above background. Using Y-50 TCR reporter cells, they formally validated its microbial-lipid specificity and, by combining lipid fractionation, mass spectrometry, and cellular assays, identified TMM as the agonistic antigen. In addition, by blocking each CD1 protein, Sakai and co-authors revealed CD1b as the TMM antigen–presenting molecule for the Y-50 TCR, which was further supported by ectopic expression of CD1b on an antigen-presenting cell line. Although Y-50 recognized TMM from different mycobacterial species, it was not reactive to the closely related Mincle ligand trehalose dimycolate (TDM) or even glucose monomycolate (GMM), a well-established *M. tuberculosis*–derived CD1b antigen. Accordingly, structure-activity relationship studies demonstrated that the trehalose disaccharide head sugar lipid moiety was required for high specificity of the Y-50 TCR to CD1b-presented TMM ligands (3).

## Structural determinants for Y-50 recognition of CD1b-TMM complexes

The presentation of lipids with bulky headgroups (akin to TMM) is often seen as poorly favorable for CD1-TCR interactions, as the headgroup can push the TCR away from the CD1-binding cleft. Nevertheless, structural evidence illustrates how particular TCRs can overcome this: headgroups can be tilted sideways along the antigen-binding cleft upon CD1 engagement by high-affinity TCRs (5, 6), or TCRs may dock onto CD1, away from the antigen-binding pocket and without engaging the lipid (7). Now, a different mechanism emerged with the crystal and cryo–electron microscopy structure analysis of the Y-50 TCR alone or bound to CD1b-TMM. Here, the unusually long Y-50 CDR3β loop displayed enough structural flexibility to move up from its unligated conformation and accommodate the complex trehalose disaccharide headgroup of TMM, allowing for ligand contact by both the TCRα and TCRβ chains in a tweezer-like mechanism (3). Indeed, this feature appeared to be key to defining antigenic specificity, as shorter CDR3β mutants failed to recognize CD1b-TMM. This strategy is reminiscent of the flexibility of CDR3β loops of the type 1 NKT TCR during CD1d lipid recognition (8).

Furthermore, Sakai and co-authors provide structural evidence supporting the idea that *TRBV4-1* germline–encoded Vβ loop residues interact with conserved amino acids in CD1b (3). These residues form a charged patch near the F′ upper antigen portal, which is conserved in CD1c, but not in other CD1 proteins, supporting the previously described *TRBV4-1* bias for both CD1b- and CD1c-restricted cells (9–11). This scenario is similar to a previously proposed role for germline-encoded Vβ loop amino acids of the type 1 NKT TCR in dictating CD1d specificity, while the hypervariable loops determine ligand specificity (8). Accordingly, several positively charged amino acids within the Y-50 CDR3α directly contacted the β-hydroxy group of MA (3), which is essential for TMM specificity, akin to CD1b-GMM recognition by different TCRs (12).

## Tracking TMM-specific T cells in peripheral blood

The development of fluorescently tagged CD1b-TMM tetramers by Sakai and colleagues represents a major technical advantage, allowing direct ex vivo characterization of a T cell subset that is shared across different individuals (3). While CD1-restricted T cells were originally thought to exhibit broad TCR diversity (13), the wider use of CD1 tetramers carrying defined ligands is expansively revealing a number of CD1 antigen–defining TCR features and biases (14–16). Now, by sorting CD1b-TMM tetramer–binding cells from multiple healthy donors, Sakai and colleagues observed the conservation of three key features alongside the Y-50 TCR: a long CDR3β chain, *TRBV4-1* bias, and positively charged CDR3α sequences (3).

Previous studies have reported that unconventional T cells (including CD1-restricted cells) typically exhibit an antigen-experienced effector phenotype with associated expression of IFN-γ, TNF, and cytotoxic molecules upon *M. tuberculosis* antigen stimulation (17). In the study by Sakai et al., CD1b-TMM tetramer^+^ T cells were almost exclusively CD4^+^, exhibiting an effector memory cytotoxic phenotype with expression of granzyme B (*GzmB*), perforin 1 (*PRF1*), granulysin (*GNLY*), *IFNG*, and *TNF* upon stimulation, akin to Y-50 (3). Frequencies of CD1b-TMM tetramer–binding T cells in healthy adults, in adults vaccinated with bacille Calmette-Guérin (BCG), or in cord blood samples were low (less than 0.005% of total T cells), but were increased in a cohort of patients with active tuberculosis (TB) disease, suggesting that mycobacterial infection drove the activation and expansion of TMM-reactive T cells in vivo. These data are consistent with previous reports of higher frequencies of CD1b-MA–restricted T cells in active TB compared with BCG-vaccinated participants (18), perhaps reflecting a requirement for prolonged antigen exposure in the proliferation or maintenance of such cell populations.

## Implications and future outlook

At present, the factors driving the CD4^+^ bias of CD1b-TMM–restricted cells remain unclear. Interestingly, CD1b-GMM– and CD1b-SGL–specific cells are also biased toward CD4 usage (14, 16). While there is no evidence that coreceptors are required for CD1b recognition, both CD4 and CD8 can modulate the functional avidity of CD1b-reactive T cells by enhancing their CD3 and TCR expression and avidity (16). Thus, CD4 expression could reflect a thymus-acquired feature during T cell selection for higher-affinity TCRs and/or be associated with a particular functional program. Interestingly, Sakai and co-authors proposed that the CD4^+^ bias may reflect a role for MHC class II in selecting CD1b-TMM–reactive TCRs (3). Whereas unconventional T cells are presumed to be selected by their cognate MHC-I–like target, a conserved subset of skin-derived, CD1a-restricted T cells has been reported to cross-react with superantigens presented by HLA-DR (19). While CD1b-TMM–reactive cells did not express PLZF (3), the key unconventional T lineage–defining transcription factor (20), PLZF^+^ CD1b-reactive T cells have been reported in CD1-humanized mice (21). It therefore remains to be understood whether the CD4^+^ and PLZF^–^ phenotypes of CD1b-TMM–reactive T cells represent a specific lineage or functional selection pathway.

In addition to clarifying the biogenesis of CD1b-TMM–reactive T cells, we can now begin to assess their antimycobacterial potential in vivo. A precise understanding of the responses that underpin protective immunity to TB disease and development of an efficacious vaccine for adults remain elusive. Despite the myriad ways in which unconventional T cells can mediate immune responses upon *M. tuberculosis* infection, a lack of knowledge of the precise antigens and responding leukocyte populations, as well as the limited availability of preclinical models have directly hampered our ability to understand their contributions to immunity against *M. tuberculosis* (17). This is particularly relevant for group 1 CD1–restricted T cells, as most rodent species lack orthologs of CD1a, CD1b, or CD1c. Consequently, nonhuman primates (NHPs) are key models for CD1 immunology. Validation of whether CD1b-TMM–restricted T cells exist in macaques will be important for the field, particularly since GMM, which is presented by CD1b in humans, is instead presented by CD1c in rhesus macaques (22). It remains unknown if the presentation of TMM or its analogs in humans and NHPs is permitted by other CD1 proteins.

A recent study showed that intravenous BCG administration is highly protective against active TB disease in NHPs (23), representing a unique opportunity to investigate correlates of protection. As the development of TNF and IFN-γ–producing CD4^+^ T cells in the airway is predictive of protection (24), it will be important to dissect the antigen specificity of these populations and determine whether they include CD1-restricted T cells. Furthermore, inclusion of various mycobacterium-derived CD1 antigens in CD1 tetramers as well as rationally designed vaccine candidates (25) and the study of humanized CD1-transgenic mice will provide new opportunities to track CD1-restricted T cell responses following immunization or mycobacterial infection (26). In particular, the ability of TMM to act as a T cell antigen and an adjuvant makes it an intriguing candidate for inclusion in novel vaccines.

Collectively, Sakai et al. (3) illustrate a successful multidisciplinary effort combining chemistry, structural and cellular immunology, bioinformatics, and mass spectrometry–based ligand discovery approaches to advance our understanding of CD1-mediated immunity. It reveals a CD1 antigen, offers insights into the molecular basis through which T cells recognize CD1b-presented targets, and highlights the multifaceted role that microbial lipids can play in stimulating both broad innate and highly specific adaptive immunity. Such advances are key to bolstering the development of new interventions to prevent and treat mycobacterial diseases.

## Version 1 03/17/2025

Electronic publication
